# Recent Advances in Nanotechnology for the Treatment of Dry Eye Disease

**DOI:** 10.3390/nano14080669

**Published:** 2024-04-12

**Authors:** Giulia Coco, Giacinta Buffon, Andrea Taloni, Giuseppe Giannaccare

**Affiliations:** 1 Department of Clinical Sciences and Translational Medicine, University of Rome Tor Vergata, 00133 Rome, Italy; giuliacoco@hotmail.it (G.C.); giacinta.buffon@ptvonline.it (G.B.); 2 Department of Ophthalmology, University “Magna Graecia” of Catanzaro, 88100 Catanzaro, Italy; andrea.taloni@studenti.unicz.it; 3 Eye Clinic, Department of Surgical Sciences, University of Cagliari, 09124 Cagliari, Italy

**Keywords:** nanotechnology, drug delivery system, dry eye disease (DED)

## Abstract

Dry eye disease (DED) incidence is continuously growing, positioning it to become an emergent health issue over the next few years. Several topical treatments are commonly used to treat DED; however, reports indicate that only a minor proportion of drug bioavailability is achieved by the majority of eye drops available on the market. In this context, enhancing drug ability to overcome ocular barriers and prolonging its residence time on the ocular surface represent a new challenge in the field of ocular carrier systems. Therefore, research has focused on the development of multi-functional nanosystems, such as nanoemulsions, liposomes, dendrimers, hydrogels, and other nanosized carriers. These systems are designed to improve topical drug bioavailability and efficacy and, at the same time, require fewer daily administrations, with potentially reduced side effects. This review summarizes the different nanotechnologies developed, their role in DED, and the nanotechnology-based eyedrops currently approved for DED treatment.

## 1. Introduction

Dry eye disease (DED) is the most common ocular surface disease, with a prevalence ranging from 5% to 50% of the adult population worldwide [1]. According to the International Dry Eye Workshop (DEWS) II, dry eye can be defined as a multifactorial disease of the ocular surface characterized by a loss of homeostasis in the tear film [2]. Tear hyperosmolarity, tear film instability, ocular inflammation, and neurosensory abnormalities are the major mechanisms involved in the disruption of this equilibrium, leading to discomfort and visual impairment [2,3]. The perpetuation and exacerbation of these conditions determine the so called “vicious cycle” of DED [4]. The DED cycle often starts with tear hyperosmolarity, due to reduced tear production and/or increased tear evaporation. Reduced tear production can be caused by either systemic autoimmune conditions, such as Sjögren Syndrome, or by any other disease leading to functional impairment of the lacrimal glands with a concomitant reduction in the aqueous layer of the tear film. Conversely, increased tear evaporation is often caused by meibomian gland dysfunction, with consequent meibomian gland failure to secrete the lipidic layer of the tear film, which physiologically prevents the evaporation of the aqueous layer underneath [5]. The resultant tear hyperosmolarity leads to ocular surface damage and increased levels of proinflammatory cytokines (IL-1β, TNF-α), proteases (MMP9), and chemokines (IL-8). The subsequent activation of the adaptive immune response with the release of IFN-γ and IL-17 also occurs [6]. Chronic inflammation determines further tear instability, amplifying the effect of tear hyperosmolarity and closing the vicious cycle. 

Dry eye disease currently represents a growing public health concern due to its impact on both visual function and quality of life, leading to a significant socio-economic burden [7,8].

The management of DED begins with control of the external environment and increased awareness of the blinking rate during several daily activities [9]. The mainstay treatment strategy to attain symptomatic relief is the use of artificial tears, preferably in their preservative-free formulations to avoid disruption of the ocular surface epithelium and the side-effects related to the frequent instillation of preservatives [10,11]. Additionally, the presence of eyelid disease must be assessed and addressed [12]. In moderate-to-severe DED, ophthalmic corticosteroids can be used to reduce inflammation, usually on a short-term basis, to avoid side effects of cataracts and glaucoma [13,14]. Other anti-inflammatory options available for long-term use include topical Cyclosporine; however, this treatment often results in poor patient compliance due to the associated ocular side effects of burning and stinging [12]. In the case of signs and/or symptoms’ persistence, further treatment options should be considered, such as prolonged therapy with topical corticosteroids, autologous serum (AS) eye drops, contact lenses, amniotic membrane grafts, or surgical punctal closure [12,15,16].

Despite multiple available treatment strategies, the effectiveness of conventional ophthalmic formulations is hampered by the presence of physiological barriers, drug dilution with tears, rapid elimination through nasolacrimal drainage, reflex tearing and blinking, protein binding, and metabolic degradation, which all contribute to reduced ocular residence time and poor bioavailability [17,18,19]. This results in less than 5% of the applied dose reaching the targeted tissues in the eye [20]. Therefore, frequent administrations and high concentrations are required to achieve and sustain therapeutic levels in ocular tissues, increasing the risk of toxicity, particularly in chronic diseases [21]. 

Enhancing the efficacy and bioavailability of ophthalmic drugs to overcome ocular barriers and prolong residence time on the ocular surface represents a new challenge in the field of ocular carrier systems [18,19,20,22,23]. Over the past few decades, research has focused on novel drug delivery systems (DDSs) based on nanotechnologies [24,25]. In fact, nanotechnology-based eyedrops offer the advantages of a prolonged ocular surface retention time, better penetration through the ocular barrier, and more targeted delivery [26,27]. Drugs delivered through nano-based delivery systems have shown enhanced adhesion to the ocular surface and reduced washout from reflex tearing and blinking, with a consequently longer retention time on the ocular surface [28,29]. Better pharmacokinetics and distribution may also contribute to lower side effects [30,31,32]. All these properties may, in turn, lead to reduced drug dosage and frequency of administration and improved patient compliance.

This review aims to highlight current advances in the development of nanotechnology formulations and their use in DED.

## 2. Methods 

**Figure 1:**
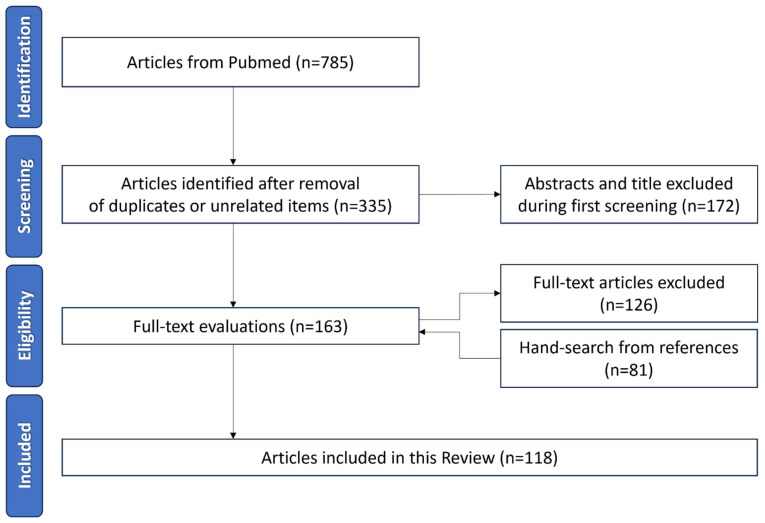
Article selection flowchart, according to the Preferred Reporting Items for a Systematic Review and Meta-Analyses (PRISMA) guidelines.

A literature search on nanotechnologies in DED was conducted on PubMed in March 2024. The search strategy used was “nano*” AND (“dry eye” OR “keratoconjunctivitis sicca” OR “eye drops”). Of the 785 articles retrieved in the search using the above terms, the articles specific to nanotechnologies in DED (n = 335) were evaluated in their abstract form. Duplicates and irrelevant papers were excluded (n = 450). English-written review articles, preclinical and clinical studies, and randomized clinical trials were included (n = 118) (Figure 1). 

## 3. Ocular Surface Drugs Barriers

The physiological characteristics of the eye reduce the bioavailability of drugs, and the main barriers for topical ocular drug delivery are represented by the tear film and the cornea [31,33].

### 3.1. Tear Film Barrier

The tear film represents the most important dynamic barrier in ocular drug delivery. It consists of an outer lipid layer, a middle aqueous layer, and an innermost mucin layer. The outer lipid layer is mainly derived from meibomian glands, with the function of reducing the surface tension, delaying tear evaporation, and preventing the overflow of tears [34,35,36]. The middle aqueous layer is produced by lacrimal glands and contains several electrolytes, proteins, and metabolites [37,38]. Finally, the inner mucin layer is mainly secreted by goblet cells in the conjunctival epithelium. It is responsible for delaying tear film rupture and protecting the cornea against pathogens.

The lipid layer acts as a barrier for lipophobic compounds, while the aqueous layer is a barrier for lipophilic ones. Additionally, the aqueous layer contains proteins and enzymes able to bind and metabolize drugs, especially in inflamed eyes [39]. Mucins are highly glycosylated proteins negatively charged that can interact electrostatically with cationic particles while repelling anionic drugs [40]. Tears are constantly secreted in the conjunctival sac, distributed on the ocular surface, and eliminated through the nasolacrimal drainage system in the nasal mucosa, where they are reabsorbed [41]. Tear secretion proceeds at a rate of 1–2 μL/min, with a total volume of about 5–10 mL [42]. The instillation of eyedrops on the ocular surface stimulates tear production and blinking reflexes, which both contribute to drug dilution and faster tear clearance. Hence, drug washout typically happens within the first few minutes after administration [43]. Furthermore, conventional eyedrops deliver a volume of 50 μL, while the eye can only accommodate about 7–30 μL, resulting in fluid spillover after instillation [44]. As a result, only 10–20% of an instilled drug remain on the ocular surface [45].

### 3.2. Corneal Barrier

The cornea represents the primary route for intraocular drug absorption. Given its multilayered structure, which combines both lipophilic and hydrophilic layers, it is relatively impermeable, and only small compounds with optimal hydrophilic/lipophilic properties are able to penetrate it. The main corneal barriers are represented by the epithelium, the stroma, and the endothelium [23].

The external lipophilic multilayered epithelium consists of 5–7 layers of epithelial cells, with tight junctions between them, and small paracellular pores of 2 nm. These pores represent a barrier to hydrophilic drugs absorbed via the paracellular pathway, while facilitating lipophilic drugs absorption via the transcellular pathway. Additionally, in physiological conditions, the presence of acidic groups on the apical surface of epithelial cells confers a negative charge, which slows the penetration of anionic particles [46,47]. The corneal stroma constitutes approximately 90% of the corneal thickness; it is hydrophilic, due to its high water content. Although being permeable to larger compounds, it presents limited penetration for lipophilic drugs [48]. Lastly, the corneal endothelium is a single layer of flat epithelia-like cells with intercellular tight junctions that act as a barrier for hydrophilic drugs. However, it represents a weaker barrier compared to the epithelium due to the lower cell thickness and larger pore size [48].

## 4. Properties of Nanoformulations

Developing effective nano-based DDSs requires a thorough understanding of their physicochemical and biological properties, with particular emphasis on particle size, surface charge, drug-loading capacity, safety, and stability.

Generally, particle size should not exceed 10 μm, since smaller particles show better stability and biodistribution [49]. Additionally, smaller particles penetrate the inner mucin layer of the tear film more rapidly; they are more easily absorbed by corneal epithelial cells and delivered into the aqueous humor, while causing less irritation. However, small particles exhibit a higher dissolution into the tear film, resulting in faster clearance [50,51,52,53].

Surface charge can impact drug delivery, influencing the stability and interaction of the particles with biological tissues. Neutral nanoparticles do not exhibit electrostatic interactions with the ocular surface, limiting their potential for adhesion, residence time, and absorption. Recently, many electrically charged particles have been developed. The zeta potential is a measure of the magnitude of the electrical charge, which can be either negative (anionic) or positive (cationic). High zeta potential values can stabilize nanoformulations thanks to electrostatic repulsion. In physiological conditions, the presence of mucins on the cornea confers a negative charge [54]. Therefore, cationic agents are attracted to the corneal surface, showing a prolonged residence time, promoting drug absorption [55]. For this reason, most nano-based formulations are prepared as cationic formulations. However, anionic particles also showed some advantages over free drugs: the repulsion between the negative charge of the particles and the negative charge of the ocular surface may reduce their adhesion, prolonging tears’ retention time [56,57]. 

Furthermore, surface morphology can influence nanoparticle distribution, cellular uptake, and toxicity. Spherical shapes improve drug performance compared to cubs or rod shapes [58,59,60].

Entrapment efficiency is another essential parameter to offer better drug protection from degradation, provide sustained drug release, and allow a high load of drugs per unit of volume. High drug loading also enables one to obtain less changes in fluid dynamics due to eyedrop instillation, thus improving biocompatibility [61].

Safety and stability should also be taken into consideration. A low level of safety may determine side effects; on the other hand, low stability may hamper efficacy due to a short shelf life. Nanocarriers are usually tested for biocompatibility, ensuring that they do not determine ocular irritation or toxicity, while being immunocompatible and biodegradable [62]. In particular, safety is usually tested by performing cytotoxicity tests in vitro and measuring the osmotic pressure generated by the particles, the pH, and other biochemical properties. The formulation should be isotonic with ocular surface tears (280–310 mOsm·L^−1^), and the pH should be approximately neutral or slightly acid (5.5–7.8) to prevent irritation [63]. Furthermore, several aspects should be evaluated to ensure the stability of the formulation: (1) chemical stability, which involves testing the resilience to various conditions such as temperature fluctuations, pH levels, and light exposure; (2) physical stability, focusing on size, shape, and distribution of the particles, which should be as reproducible and consistent as possible; and (3) compatibility of the nanoparticles with other components of the eye drops, to identify undesirable interactions between agents [64].

## 5. Nano-Based Drug Delivery Systems in Dry Eye Disease

Eye drops represent the most widely used and readily available formulations for DED treatment. However, reports indicate that 90% of the eye drops available on the market only achieve 5% drug bioavailability. Most of the drug is removed through tear fluid and enzymatic degradation or may not be absorbed due to the physiological eye barrier [33]. 

Many attempts have focused on the fabrication of multi-functional nanosystems such as nanoemulsions, liposomes, dendrimers, hydrogels, and other nanosized carriers as effective alternatives to conventional eye drops in ocular disease therapy. In this scenario, nano-based DDSs have been shown to enhance adhesion to the ocular surface, minimizing drug washout due to tearing and blinking behavior. This effect can prolong the residence time of drugs on the ocular surface, while also improving the drug’s ability to cross the ocular barrier and reach its target. As a result, these systems enhance the bioavailability and efficacy of the drug [65]. Many nano-based DDSs with distinct and specific characteristics have been developed so far [66].

### 5.1. Nanoemulsions

Nanoemulsions are made of a two-phase system of water, oil, and amphiphilic surfactants featuring nanoparticles within a size range of 10 to 100 nanometers. After instillation, the water phase of the emulsion has the potential to boost the aqueous layer of the tear film, providing moisture to the cornea. Upon the breakdown of oil droplets, the encapsulated emulsion components are released. Subsequently, the oil phase integrates with the natural lipid layer, fortifying it and minimizing fluid loss due to evaporation. Emulsifiers can be used to increase mucus layer depth and enhance the “wettability” of the tear film [67].

Because of their globule size, nanoemulsions are often thermodynamically unstable and require a high concentration of surfactant to stabilize their structure, with a subsequent risk of intolerance. On the other hand, the use of cationic surfactants may prolong drug bioavailability on the ocular surface thanks to the electrostatic interactions with the corneal epithelium. Thanks to their composition, nanoemulsions can interact with the lipid layer of the tear film persisting in the conjunctival sac for an extended period of time, thereby serving as a reservoir for drug release over time [68].

Cyclosporine A (CsA) is commonly prescribed as an immunosuppressant for DED treatment. It inhibits the activation of T lymphocytes and prevents the mitochondria-mediated apoptosis pathway. However, due to its hydrophobic nature, CsA has poor aqueous solubility, requiring a specific nanocarrier to enhance its bioavailability on the ocular surface [69]. By leveraging emulsion proprieties, a 0.05% preservative-free (CsA) anion oil-in-water nanoemulsion was the first CsA formulation approved by the Food and Drug Administration (FDA) in 2003 for DED treatment [70].

### 5.2. Nanomicelles and Polymeric Micelles

Micelles are colloidal structures that spontaneously form in a solution when the concentration of the surfactant or polymer exceeds the critical micellar concentration. Nanomicelles are nanosized (10 to 200 nm) colloidal carrier systems characterized by a hydrophobic core and a hydrophilic shell that self-assemble in aqueous solutions. These amphiphilic copolymers are commonly used as pharmaceutical vehicles in ocular tissues, and their structure can be adapted to obtain specific proprieties [71]. For instance, residence time on the ocular surface can be improved by introducing a cationic charge to interact with the negatively charged mucins on the ocular surface or by adding reactive groups, such as thiol groups, to bind the chemical moieties present in the tear fluid [72].

Polymeric nanomicelles are created by synthesizing block copolymers that contain distinct hydrophobic and hydrophilic monomer units [73]. On the other hand, nanosized micelles formed by amphiphilic molecules, featuring water-attracting head groups and hydrophobic tails, are defined as surfactant nanomicelles [74].

The mucoadhesive nature of nanomicelles allows for enhanced interaction with the ocular surface, while their small size facilitates tissue penetration. Furthermore, due to their high water solubility, nanomicelles produce clear aqueous solutions that can be easily used in the form of eye drops without causing any interference with vision [75].

The solubility of hydrophobic drugs can also be increased using micelles. In 2018, Yu Y. et al. carried out in vitro and in vivo studies to demonstrate how micelle formulation can improve the bioavailability and solubility of CsA, achieving a longer and enhanced effect against ocular surface diseases [76]. In 2019, Mandal et al. conducted in vivo studies on loaded octoxynol-40 micelles, demonstrating a statistically significant improvement in ocular surface parameters after both single- and multi-dose administrations over 5 days [77].

### 5.3. Nanosuspensions

Nanosuspensions are colloidal dispersions in which drug particles are reduced to the nanometer scale and dispersed in a liquid medium, typically water or another solvent, to enhance the dissolution and bioavailability of poorly water-soluble drugs. Various methods, including high-pressure homogenization, media-milling, and precipitation techniques, can be employed to create nanosuspensions. The stabilizers used in the formulation of ophthalmic nanosuspensions often consist of natural, synthetic, or hybrid polymers [78]. However, the application of nanosuspensions for treating DED is constrained by physical instability issues, such as sedimentation, and potential toxicity arising from the use of surfactants [72]. Eudragit, a biocompatible polymer derived from polymethacrylate, is frequently utilized in the preparation of nanosuspensions to stabilize their structure, leading to prolonged drug release times and heightened efficacy. This highlights the beneficial impact of altered surface proprieties on nanosuspensions to improve bioavailability and drug release times [79].

Nanosuspension technology offers a secure and efficient method of delivering hydrophobic drugs to the ocular surface. However, like nanoemulsions, enhancing the physical stability of these nanocarriers is crucial for their practicality [66,78]. For instance, in the study conducted by Wu et al., the chitosan-modified mycophenolate mofetil nanosuspension showed decreased drug clearance compared to the non-chitosan-modified nanosuspensions [80]. 

### 5.4. Liposomes, Niosomes, and Cubosomes 

Lipid-based formulations have long been studied to create biocompatible nanocarriers, since cell membranes consist of lipids. 

#### 5.4.1. Liposomes

Liposomes are vesicular systems composed of one or more concentric phospholipid bilayers separated by an aqueous buffer. They allow the encapsulation of both hydrophobic and hydrophilic drug molecules, respectively, in the lipid bilayer and in the aqueous compartment. This amphiphilic structure protects drug molecules from degradation by enzymes on the ocular surface and makes liposomes a suitable drug-delivery system in both anterior and posterior chambers [81].

Many drugs have been formulated using a liposomal approach for ocular use, and most of them are already on the market for DED treatment. 

By mimicking cell membrane architecture, liposomes achieve high biocompatibility and drug-loading capacity. Furthermore, the possibility of changing their characteristics thanks to different compositions of lipids, surface charges with cationic molecules, size of vesicles, or method of preparation makes them a suitable DDS to target different ocular tissues [82]. 

The positive superficial charge of liposomes facilitates interactions with the negatively charged mucin layer in the tear film, particularly when coated with adhesive polymers or dispersed into an adhesive gel to enhance cornea binding [82]. In 2021, Lopéz-Machado and colleagues used the anti-inflammatory and antioxidative properties of lactoferrin, a glycoprotein endogenous in ocular tissues, to create a hyaluronic acid (HA)-coated lactoferrin liposome [83]. Pharmacokinetic and pharmacodynamic profiles were evaluated both in vitro and ex vivo showing prolonged stability, permeability, and bioavailability, with the amelioration of DED symptoms and without any sign of cytotoxicity [83]. 

#### 5.4.2. Niosomes

Niosomes are vesicular DDSs composed of non-ionic surfactants and cholesterol, able to form spontaneous solutions when surfactants and cholesterol are hydrated. Like liposomes, niosomes are made of a lipid bilayer that allows them to encapsulate both hydrophilic and hydrophobic drugs; however, they are structurally different from liposomes due to the absence of phospholipids. Several studies indicate that these nanostructures, thanks to their composition, can open the tight junctions and modify their corneal permeability properties, enhancing the bioavailability and therapeutic efficacy of drugs in the target tissue [84].

Tacrolimus, an immunosuppressant drug, is currently under investigation for DED treatment owing to its capacity to suppress the immune response by inhibiting the release of inflammatory cytokines [85]. In 2016, Zeng W. et al. developed HA-coated niosomes to enhance the transcorneal permeability and therapeutic efficacy of tacrolimus. The improvement in aqueous humor was significant, demonstrating a 2.3-fold increase compared to tacrolimus suspension [86]. 

#### 5.4.3. Cubosomes

The other lipid vesicular systems adopted as nanocarrier DDSs are cubosomes, nanoparticles with a diameter between 100 and 300 nm and a cubic liquid crystalline phase. Cubosomes are produced with specific amphiphilic lipids in the presence of an appropriate stabilizer that allows the encapsulation of hydrophobic, hydrophilic, and amphiphilic compounds. The integration of cubosomes with other emerging technologies such as gelation, surface coating, and polymer incorporation may enhance the efficacy and long-lasting action of these biocompatible nanocarriers [72].

### 5.5. Polymeric Nanoparticles, Solid Lipid Nanoparticles, and Nanostructured Lipid Carriers

Nanoparticles are minuscule particles of a nanoscale size (range: 10–100 nm) characterized by both biodegradability and a composition of colloidal polymers [87]. 

#### 5.5.1. Polymeric Nanoparticles

Depending on the preparation method, polymeric nanoparticles loaded with drugs can take the form of nanospheres, where the drug is uniformly dispersed throughout the polymer matrix, or nanocapsules, where the drug is encapsulated within the polymer shell. In order to improve the adherence of nanoparticles to the negatively charged ocular surface, their matrix can be coated or conjugated with a wide range of positively charged polymers. One of the most employed polymers is chitosan, a polysaccharide derived from chitin, able to prolong drug residence time on the ocular surface thanks to its high biocompatibility and positively charged nanoparticle surface. In vivo studies have demonstrated that chitosan nanoparticles can extend the release of Cyclosporin A and enhance its penetration into the ocular surface [88]. 

Recently, polymeric nanoparticles loaded with tacrolimus for the treatment of DED were designed using ionotropic gelation with the natural polymer gellan gum. These nanoparticles demonstrated increased precorneal retention and sustained drug release [89]. Additionally, in a rabbit model, treatment with tacrolimus nanoparticles resulted in a reduction in the symptoms of DED [90].

#### 5.5.2. Solid Lipid Nanocapsules and Nanostructured Lipid Nanoparticles

Solid lipid nanocapsules (SLNs) are nanoscale structures made of a lipid core, in a solid state at room temperature, which provides a stable matrix for drug encapsulation, surrounded by a phospholipid layer which contributes to the stability and biocompatibility of the nanocapsules. This unique structure offers advantages such as controlled release, reduced immune reactions, and protection of the active molecules from degradation, resulting in enhanced residence time on the ocular surface [82].

The second generation of lipid nanoparticle technology is represented by nanostructured lipid nanoparticles (NLCs), with at least 30% triglycerides in a liquid state at room temperature. The addition of liquid lipids (oils) allows for a higher loading capacity of encapsulated drugs and, notably, reduces the risk of drug expulsion during storage [91]. Thus, both SLNs and NLCs are efficient systems for ocular drug delivery, and the incorporation of a liquid lipid into the matrix of NLCs guarantees enhanced physical stability to these carriers [92].

The invention of newer solid lipid nanoparticles and nanostructured lipid carriers marked a significant breakthrough in the field of nano-DDSs, offering increased stability, enhanced specificity in site delivery, and a reduction in immune reactions [93]. An experimental cysteine-nanostructured lipid carrier was synthesized for the topical administration of Cyclosporine A. This formulation exhibited a prolonged retention time in aqueous humor, tears, and eye tissues compared to an oil solution, due to the bioadhesive properties and sustained-release characteristics of NLCs [94]. Therefore, when tested as an artificial tear film in a rabbit evaporative dry eye model, NLCs demonstrated remarkable efficacy in protecting the corneal surface against desiccating stress [95].

In 2019, Yu and colleagues developed a water-soluble cerium oxide-loaded glycol chitosan nanoparticle as a new type of eye drop and tested it in a murine model of DED [96]. The evaluation revealed a significant improvement in the tear film break-up time test and tear volume and a decrease in intracellular reactive oxidative species levels in the mice cornea and conjunctiva. These results underscored the efficacy of these nanoparticles as efficient DDSs and their potential in controlling inflammation levels and treating DED [96].

Nanocapsules, consisting of an oil core within a polymeric shell, can also be employed to achieve better drug loading in the delivery of lipid-soluble drugs. In an in vivo rabbit model, Zhang A et al. encapsulated CsA within lipid nanocapsules to create eye drops for DED treatment. Their study demonstrated a significant improvement in CsA bioavailability, along with a safe profile, resulting in enhanced therapeutic effects in a rat model of DED [97].

### 5.6. Nanowafers 

The term “nanowafers” refers to nanostructures composed of biodegradable and biocompatible polymers loaded with drugs with a thin and flat design. Nanowafers act as drug reservoirs that can be easily applied on the ocular surface, releasing the drug until biodegradation. They are structured as nanosized transparent membranes or discs that facilitate drug absorption into anterior ocular tissues and protect the corneal surface [72].

Nanowafers represent another novel modality in ocular drug delivery for DED. They extend the contact time of the drug with the ocular surface and serve as protective polymer membranes, aiding in the healing of injured corneas, commonly associated with DED [20]. In a study by Bian F. et al., a dexamethasone-loaded nanowafer was developed for the treatment of DED and tested for its efficacy in a mouse model. Following the treatment period, the dexamethasone nanowafer demonstrated the ability to restore corneal barrier function and reduce the overexpression of inflammatory cytokines [98].

### 5.7. Dendrimers

Dendrimers are nanoscale macromolecules characterized by a tree-like or dendritic structure, featuring highly branched repeating units radiating from a central core. The three-dimensional shape and size of the dendrimer are determined by the number and arrangement of arms comprising its core, while its physiochemical proprieties are dictated by the surface groups [99].

The star-shaped multi-branched structure of dendrimers enables them to encapsulate a large number of lipophilic or hydrophilic drugs, and their potential for surface modification enhances their versatility as nanocarrier systems. Vandamme et al. conducted comprehensive investigations into the corneal residence time of polyamidoamine dendrimers using an in vivo rabbit model [100]. Their observations revealed a substantial impact of both the dendrimer size and its terminal groups on controlled ocular drug delivery. In particular, they hypothesized that larger dendrimers with hydroxyl terminals may exhibit prolonged corneal residence times and improved efficacy, suggesting an interaction with ocular mucins as a contributing factor [100]. An alternative approach to harness topically applied dendrimers involves integrating them in in situ polymerizing gels to extend the corneal residence time and improve drug delivery efficacy [101].

Ocular surface inflammation is frequently observed as a key pathogenic event in DED. Numerous nanocarriers of corticosteroids, such as dexamethasone, have extensively been studied to address manifestations of ocular inflammations while minimizing potential side effects. In 2017, Soiberman et al. developed a subconjunctival injectable gel based on dendrimers and HA incorporated with dexamethasone [102]. The efficacy of this formulation was evaluated in a rat model, demonstrating reduced corneal thickness and inflammation compared to a free dexamethasone formulation. By specifically targeting inflammatory cells, the dexamethasone dendrimer gel improved corneal clarity without causing an increase in intraocular pressure when compared with free dexamethasone [102].

### 5.8. In Situ Hydrogels

Hydrogels are made of a three-dimensional structure of hydrophilic polymers such as HA, chitosan, and methylcellulose, which enable them to absorb a significant amount of water without dissolving. This crosslinked matrix allows them to encapsulate a wide range of hydrophobic or hydrophilic drugs and may also be customized in various shapes and thicknesses for drug delivery across the ocular surface. 

In situ hydrogels can undergo a sol–gel transformation from a liquid to a semi-solid or solid state, triggered by various stimuli, including temperature, pH, or ions on the ocular surface. Eye drops made of thermoresponsive hydrogels remain in a liquid state at room temperature and undergo a sol–gel transition upon administration, triggered by the ocular surface temperature surpassing the low critical solution temperature. Their adjustable physical characteristics and degradation rates provide spatial and temporal control over the environment, prolonging drug retention time, targeted site delivery, and therapeutic efficacy [103]. 

In 2021, Yu Y. and colleagues developed a synthetic soft hydrogel containing HA as a long-acting ocular surface lubricant for treating DED [104]. The crosslinked HA hydrogel showed high biocompatibility in a canine clinical study, leading to significant improvement in ocular surface signs and symptoms of dry eye [104].

### 5.9. Drug-Eluting Contact Lenses

To extend drug contact time on the ocular surface, contact lenses are engineered using polymeric materials, such as hydrogels. These encapsulate drug molecules leading to increased absorption in the ocular tissues and reduced drug loss via the tear ducts. Typically, drugs are loaded in contact lenses by soaking; other techniques include nanocarriers, molecular imprinting, drug-infused ring implant, or direct incorporation into the contact lens matrix [105]. Regardless of the method employed, ocular lenses are designed to comfortably fit on the cornea through physical adherence or surface tension and must preserve transparency and oxygen permeability.

Conventional hydrogels and silicone hydrogels are two major materials employed in the fabrication of soft contact lenses designed for drug elution. In a study conducted by Maulvi FA et al., two methods for loading HA into hydrogel contact lenses were proposed: the soaking method and direct entrapment [106]. In both cases, cytotoxicity studies indicated a favorable safety profile for hydrogel contact lenses. Furthermore, in vivo measurements in rabbit tear fluid demonstrated an increased residence time of HA with lenses compared to conventional eye drop treatments [106].

Employing these techniques, anti-inflammatory drugs such as dexamethasone, betamethasone, and Cyclosporine A have been incorporated into contact lenses, increasing drug contact time on the ocular surface and, thereby, therapeutic efficacy [107,108].

### 5.10. Nanogels

Nanogels are amongst the most recent nanotechnologies studied for drug delivery to the ocular surface. They combine nanoparticles and hydrogel properties to create a three-dimensional crosslinked polymeric network. Such structure can incorporate small molecules and, thanks to the hydrogel, offer high ocular bioavailability, drug-loading capacity, and biocompatibility. They can be categorized based on the type of bonds in the polymer network. Physically crosslinked nanogels are characterized by non-covalent interactions. These nanogels are easy to prepare; however, they are fragile and unstable due to the low binding energy. Conversely, chemically crosslinked nanogels are made of covalent bonds that allow a higher stability, but they have a higher toxicity risk due to crosslinking agent residues after preparation [109]. 

“Smart nanogels” are a new promising strategy based on sensitivity to chemical, physical, or biological stimuli to control drug delivery and release. Thermosensitive polymers such as polymer N-isopropylacrylamide (PNIPAAM) have been used to create temperature-responsive nanogels: a sol–gel transition upon temperature changes leads to drug release, increasing ocular bioavailability and drug retention time on the ocular surface [110]. 

Lin et al. [111] used the controlled pyrolysis of lysine hydrochloride to create lysine-carbonized nanogels, which showed high biocompatibility in both in vivo and in vitro experiments [111]. This nanogel showed antioxidant, anti-inflammatory, and bioadhesive properties, which makes it a potential future DDS for the long-term treatment of DED [111].

### 5.11. Nanozymes

Nanozymes are novel nanomaterials that mimic the kinetics and activity of natural enzymes by catalyzing the reactions of substrates, like in physiological conditions. Nanozymes have active sites where the catalysis of the reaction occurs. The catalytic centers usually consist of single or multiple metal atoms inside the nanozymes. Recent advancements in the production of nanozymes allowed for a very high selectivity, optimized atomic utilization rate, and improved catalytic activity [112]. Zou et al. developed a cerium oxide nanozyme combined with branched poly(ethylene imine)-graft-poly(ethylene glycol) for the treatment of DED. It mimics the activity of superoxide dismutase and catalase to scavenge reactive oxygen species (ROS). This nanozyme has a positive surface charge, which facilitates endocytosis by human corneal epithelial cells. Furthermore, the cerium oxide nanozyme has demonstrated antioxidant properties, both in vitro and in vivo, ameliorating corneal epithelial defects and increasing goblet cell number in a dry eye murine model [113]. More recently, Chu et al. produced dual-atom nanozyme (DAN) eye drops based on Fe and Mn atoms embedded in N-doped carbon material modified with a hydrophilic polymer [112]. This formulation is designed to inhibit NLPR3 inflammasome activation and neutralize ROS, reducing inflammation in patients with DED. The researchers assessed the antioxidative, anti-apoptotic, and anti-inflammatory properties in human corneal epithelial cells. DAN effectively reduced ROS, oxidative DNA damage markers, and the levels of proinflammatory cytokines. The formulation was also tested on a murine dry eye model to assess in vivo therapeutic efficacy and safety. Corneal opacity and fluorescein staining were significantly reduced, while the tear volume was significantly higher compared to the control group (0.05% CsA). No safety concerns emerged during the study [112].

**Figure 2:**
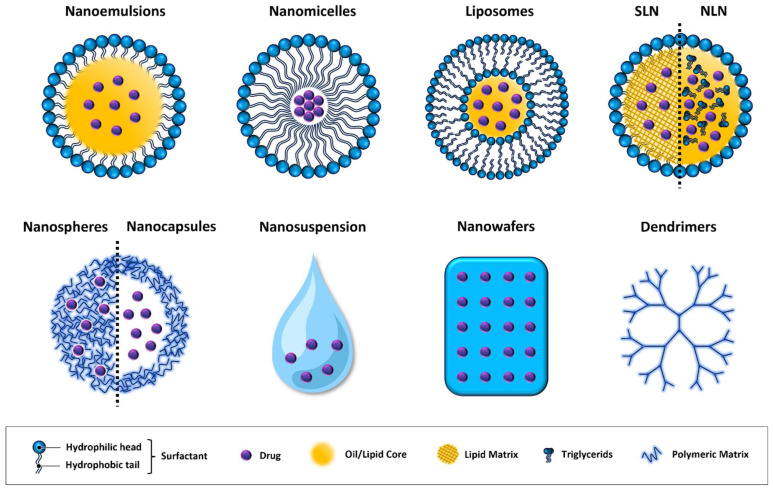
Graphic representation of drug delivery systems. SLN = solid lipid nanocapsules; and NLN = nanostructured lipid nanoparticles.

Figure 2 depicts the different nanomaterials used for drug delivery; Table 1 summarizes the key features of the nanotechnologies presented in this review; and Table 2 shows studies on nanotechnologies in DED.

## 6. Nanotechnologies Currently Approved for Dry Eye Disease

Studies both in vitro and in vivo have demonstrated that novel drug delivery nanosystems represent a potential new strategy in DED treatment, offering distinct advantages over conventional palliative therapy with lubricant eye drops. Several ocular nanocarriers are undergoing clinical trials or are at various stages of development, while many others have already received FDA approval and are available on the market.

Restasis^®^ was the first marketed nanoemulsion approved by the FDA in 2002 for the treatment of dry eye. Restasis^®^ is composed of a 0.05% oil-in-water anionic nanoemulsion of CsA with polysorbate 80 as a surfactant and castor oil as a solubilizer [74]. Phase 3 clinical trials involving patients with DED treated with a CsA 0.05% ophthalmic emulsion demonstrated an increase in conjunctival goblet cell density and significant reductions in both punctate fluorescein staining and the symptoms of blurred vision. Additionally, no significant topical or systemic side effects were recorded, highlighting the efficacy and the favorable safety profile of the CsA emulsion in the treatment of DED [124,125]. Similarly, Lacrinmune^®^ is a nanoemulsion of CsA available as an ophthalmic formulation, akin in composition to Restasis^®^, with the added inclusion of sodium hyaluronate to improve precorneal residence time [70].

The Novasorb^®^ technology was developed to leverage the electrostatic attraction between cationic formulations and the negatively charged ocular surface, resulting in an extended ocular residence time of drugs [126]. In addition, the nanosize of oil droplets can enhance the stability of the emulsion and improve ocular absorption. By employing the Novasorb^®^ technology, products such as Cationorm^®^ and Ikervis^®^ have been developed as nanoemulsion formulations for managing DED symptoms, allowing improved ocular tolerability and higher CsA bioavailability compared to Restasis^®^ [127,128].

Another nanoemulsion formulation based on the Novasorb^®^ technology is Cyclokat^®^, a cationic emulsion of CsA at 0.1%. The Sansika study, a phase III trial, assessed the efficacy of Cyclokat^®^ by demonstrating the impact of this cationic formulation on patients with severe DED [129].

Although nanoemulsion technology has led to improved persistence on the ocular surface, its use is restricted because of stability issues related to aggregation and some ocular adverse effects. These include instillation site pain and toxicity with long-term use [130,131].

In recent years, novel DDSs have been employed to develop new formulations of CsA aimed at mitigating ocular side effects and achieving prolonged therapeutic effects. One such formulation is OTX-101, marketed as Cequa^®^, which is a nanomicellar formulation containing 0.09% CsA [132]. A comparative study between Restasis^®^ and Cequa^®^ demonstrated a significantly higher CsA concentration in ocular tissues after a single topical administration of the OTX-101 nanomicellar formulation compared to the CsA nanoemulsion [133]. Furthermore, phase III confirmatory clinical trials reported only a few mild cases of side effects such as instillation site pain and hyperemia, similar to other drugs already approved in the category, highlighting the safe profile of this formulation [134]. As a result, Cequa^®^ received FDA approval in 2018 for treating the signs and symptoms of DED [77].

Numerous drugs have been developed using a liposomal approach for ocular applications, with many of them already on the market for the treatment of DED. Liposome-based ocular products, such as Vyseo^®^, Clarimist^®^, and Tears Again^®^, have demonstrated effectiveness in enhancing tear fluid stability and reducing tear fluid osmolarity. They are suitable for treating patients with mild-to-moderate evaporative DED [74]. Specifically, Tears Again^®^ (currently marketed in the UK as Optrex ActiMist^TM^) is a phospholipid liposomal spray that can be applied to the closed eyelids, enabling the liposomes to migrate through the lid margin into the tear film. Studies have shown that a single application of this phospholipid liposomal spray can result in significant improvements in tear film stability and lipid layer thickness lasting between 60 and 90 min [135]. In a comparative study, Tears Again^®^ exhibited superior ocular comfort and increased tear stability compared to two other liposomal sprays available on the market [136]. This difference has been attributed to the ability of phosphatidylcholine, when delivered in a stable liposomal form, to migrate across the eyelid margins and integrate with the tear film, thereby enhancing its stability [136].

Given the frequent association of vitamin deficiencies with DED, vitamin supplementation can be beneficial in ameliorating the signs and symptoms in individuals suffering from dry eye [137]. Extensive preclinical evidence suggests that vitamin deficiencies correlate with cell degeneration, nerve damage, and reduced tear film quality [138]. Vitamin deficiencies initially impact goblet cells, followed by epithelial cells and meibomian glands, leading to impaired wound healing and heightened oxidative stress. The topical administration of vitamins, facilitated by liposomal delivery to enhance bioavailability, has the potential to counteract these processes and aid in managing manifestations of DED. Products such as Lacrisek^®^ (vitamin A palmitate and vitamin E liposomal spray) and Artelac Rebalance^®^ (vitamin B12 liposomal eye drops) demonstrated to improve the signs and symptoms in patients with DED and received FDA approval.

An alternative method to prolong the ocular residence time has been devised by formulating mucus-penetrating nanoparticles. This technology involves specific nanocarriers designed with an engineering coating that prevents adherence to mucins and allows effective mucus penetration [139]. This approach was utilized to produce 0.25% loteprednol etabonate nanoparticles coated with Poloxamer 407, marketed as Eysuvis^®^ (KPI-121 0.25%), which received FDA approval in 2020 for the short-term (up to two weeks) treatment of DED [70]. In preclinical trials, KPI-121 0.25% demonstrated good tolerability and a good safety profile, with no notable differences observed in intraocular pressure at the end of the 2-week treatment. Therefore, KPI-121 0.25% can be considered a viable option for the short-term therapy of DED, particularly in patients with a more pronounced inflammatory component [140].

Recently, ocular DDSs based on hydrogel nanotechnology have garnered significant research attention. Various hydrogel formulations such as Vidisc^®^ gel, Hylo^®^gel, GelTears^®^, Viscotears^®^, and Clinitas gel^®^ have obtained FDA approval and are now commercially available as treatments for DED [120]. The ability to encapsulate a broad spectrum of hydrophilic and hydrophobic drugs, coupled with excellent biocompatibility and sustained drug release on the ocular surface, make hydrogels a promising technology for addressing ocular surface disease [82].

One of the most recent nanotechnologies approved for treating DED is Cyclasol 0.1% (Vevye TM Cyclosporine ophthalmic solution 0.1%), which is a preservative-free nonaqueous formulation of CsA [141]. The higher concentration of CsA and the lack of preservatives enhance the bioavailability and efficacy of Cyclasol 0.1%, surpassing even those of Restasis^®^ and Ikervis^®^ [16].

All these nanoformulations highlight the significant progress achieved in the design of nanotechnology-based approaches aimed at overcoming the limitations of ophthalmic formulations for managing DED. Many other drugs have already progressed through several steps of their development process, while others are still in the preclinical phase. Table 3 lists the nanotechnologies currently approved for DED.

## 7. Conclusions

Research on nanotechnology for DED treatment has progressed considerably in recent years, focusing on the development of new strategies for nanocarrier delivery systems. Such innovative DDSs are designed to enhance drug penetration through ocular barriers and increase drug bioavailability and efficacy. The capacity to deliver a wide range of drugs and a customizable structure that can respond to changes in the ocular microenvironment are two significant qualities of these novel DDSs.

Several in vitro and in vivo studies have demonstrated that these emerging DDSs represent a potential new strategy in DED treatment. They offer distinct advantages over conventional therapy, while showing good safety and a good ocular toxicity profile. Based on their similarity to the three-layered tear film, various lipid-based nanocarriers including micelles, liposomes, and nanoemulsions were found to enhance drug availability on the ocular surface. Emulsions with an oil-in-water composition can transport both hydrophilic and hydrophobic drugs through the oil droplets, simultaneously providing moisture to the cornea via the water phase [153]. Moreover, the self-gelling behavior of polyoxyethylated non-ionic surfactant may be used for the development of a thermosetting ophthalmic DDSs able to pass through the tight junctions and inhibit the glycoprotein P on the epithelial cells, resulting in an enhancement of the corneal transport of the drug included in the droplet [154].

Despite the promising impact of nanocarriers as new treatment options, the translation of nanotechnologies in clinical practice faces several challenges. On the one hand, the manufacturing of nano-based DDSs is complex and expensive; achieving a consistent product quality is paramount to ensuring effectiveness. In certain cases, this may eventually lead to scalability issues, with limited drug production. Additionally, there is a lack of uniform international standards for the production and testing of nanomaterials. Given the limited knowledge about this subject, quality assessments are often performed on a case-by-case basis, which inevitably causes regulatory hurdles and longer approval times [155]. Although some nano-based formulations have already received regulatory approval and are currently available on the market, more extensive studies on humans are required to obtain more robust data on their efficacy, ocular toxicity, and biocompatibility. Further improvements in the design and performance of nano-based DDSs are still required.

To date, there are no curative treatments for DED; clinicians primarily focus on symptoms’ management and improving tear film quality to provide temporary relief. This unmet need underscores the priority of developing more effective therapeutic strategies. Nanotechnology may pave the way for more targeted treatments, with improved penetration, bioavailability, and efficacy. New molecules may be developed in combination with nanocarriers to optimize their mechanism of action. At the same time, nano-based DDSs may not exclusively affect the clinical outcome but also the quality of life of the patients, by requiring a lower instillation frequency and causing less side effects. In conclusion, nanotechnology-based formulations are expected to change the status quo in ocular DDSs, especially for anterior-segment eye diseases such as DED.

## Figures and Tables

**Table 1:** Composition, advantages, and disadvantages of nanotechnologies applied to dry eye disease.

Nanotechnology	Composition	Advantages	Disadvantages
Nanoemulsions [67,68]	Two-phase system of water, oil, and amphiphilic surfactants. The oil phase integrates with the lipid layer of the tear film, while the water phase integrates with the aqueous layer and emulsifiers with the mucous layer.	Long residence time, can be used as reservoir for slow drug release.	Thermodynamic instability. Potential intolerance due to high surfactant concentration.
Nanomicelles [71]	Hydrophobic core and hydrophilic shell. Employed to encapsulate, solubilize, and deliver hydrophobic drugs.	High water solubility, forming clear aqueous solutions which do not cause vision blurring.	Potential stability issues.
Nanosuspensions [78]	Colloidal dispersions where drug particles are reduced to the nanometer scale and dispersed in a liquid medium.	Enhances bioavailability of poorly soluble drugs.	Physical instability issues (sedimentation) and potential toxicity due to the use of surfactants.
Liposomes [81,114,115]	One or more concentric phospholipid bilayers separated by an aqueous buffer, which allow the encapsulation of both hydrophobic (in lipid bilayer) and hydrophilic (in aqueous compartment) drug molecules.	High biocompatibility. Protects drug molecules from enzymatic degradation. Encapsulate both hydrophobic and hydrophilic drugs.	Potential risk of aggregation and fusion. Limited stability in storage.
Niosomes [86]	Structurally different from liposomes due to the absence of phospholipids. Composed of non-ionic surfactants and cholesterol.	Encapsulate both hydrophilic and hydrophobic drugs.	Structurally different from natural membranes for the absence of phospholipids. Stability concerns in aqueous environments.
Cubosomes [116]	Composed of specific amphiphilic lipids in the presence of an appropriate stabilizer to form cubic liquid crystalline phase	Suitable for hydrophobic, hydrophilic, and amphiphilic compounds.	Complex manufacturing process. Stability issues related to the crystalline phase.
Polymeric Nanoparticles [117]	Depending on the preparation method can form nanospheres (drug is uniformly dispersed throughout the polymer matrix) or nanocapsules (drug is encapsulated within the polymer shell).	Potential for targeted delivery to specific tissues.	Complex manufacturing process. Potential toxicity due to polymers.
Solid Lipid Nanoparticles (SLNs) [87]	Structures made of a lipid core, in a solid state at room temperature, that provides a stable matrix for drug encapsulation and a surrounding phospholipid layer that contributes to stability and biocompatibility.	Controlled drug release. Protects active molecules from degradation. Good biocompatibility and safety profile.	Poor drug-loading capacity and drug expulsion after polymeric transition during storage and relatively high water content of the dispersions.
Nanostructured Lipid Carriers (NLCs) [94,95]	Similar to SLNs. They include at least 30% triglycerides in a liquid state at room temperature.	Controlled drug release. Protects active ingredients from degradation. Enhanced physical stability. Higher drug-loading capacity.	Complex manufacturing process. Potential drug leakage.
Nanowafers [118]	Biodegradable polymers loaded with drugs, with a thin and flat design.	Extended drug contact time. Protect corneal surface.	Potential discomfort upon application.
Dendrimers [119]	Tree-like or dendritic structure, featuring highly branched repeating units radiating from a central core. Star-shaped multi-branched structure enables it to encapsulate a large number of lipophilic or hydrophilic drugs.	High drug encapsulation efficiency. Controlled drug release.	Complex manufacturing process. Potential cytotoxicity.
In Situ Hydrogels [120]	Three-dimensional structure of hydrophilic polymers (hyaluronic acid, chitosan, and methylcellulose) that can absorb a significant amount of water without dissolving.	Responsive to environmental stimuli. Prolonged drug retention. Customizable in various shapes and thicknesses.	Variable sol–gel transition rates. Potentially inconsistent drug release.
Drug-eluting Contact Lenses [105]	Contact lenses engineered using polymeric materials, such as hydrogels, which encapsulate drug molecules.	Extended contact with the ocular surface. Increased drug absorption. Reduced drug loss via tear ducts.	Need for lens compatibility. Risk of lens-related complications.
Nanogels [109]	Three-dimensional crosslinked polymeric network. Categorized based on the type of bonds in the polymer network (non-covalent, covalent).	Easy manufacturing process. High drug-loading capacity. Smart nanogels are thermosensitive.	Fragility of physically crosslinked nanogels. Potential toxicity in chemically crosslinked varieties.
Nanozymes [112]	Nanozymes mimic natural enzymes’ activity. The active sites for the catalysis of the reaction usually consist of single or multiple metal atoms.	Mimic a naturally occurring process. High selectivity.	More studies are necessary to assess tolerability.

**Table 2:** Nano-based formulations under study in dry eye disease.

Category	Drug	Nanosystem	Study Model	Outcomes	References
Emulsions	Cyclosporine A	Emulsion of glycerin, castor oil, polysorbate 80, carbomer copolymer A	In vivo (animal and humans)	-Improved dry eye symptoms and signs	Ames P. et al. [121]
	Tacrolimus	Microemulsion prepared by titration with propylene glycol and polysorbate 80	In vitro and in vivo (rabbit model)	-Increased drug penetration -No toxicity to corneal and conjunctival cells	Silva-Cunha A. et al. [122]
Micelles	Cyclosporine A	Methoxy poly (ethylene glycol)-poly (lactide) polymer (mPEG-PLA) micelles	In vitro and in vivo	-Stability for at least 3 months and sustained release -Enhanced retention time with a longer effect toward DED symptoms	Yu Y. et al. [76]
	Cyclosporine A	HCO-40/OC-40 based non-ionic nanomicelles	Preclinical and clinical trials	-Highly effective and safe -Rapid onset of action	Mandal A. et al. [77]
Nanosuspensions	Mycophenolate Mofetil	Chitosan-modified nanosuspensions	In vivo (rabbit model)	-Increase corneal mucoadhesion and drug absorption -Prolonged survival time of high-risk allografts -Inhibition of corneal immune rejection in the rabbit models of penetrating keratoplasty	Wu XG et al. [80]
Liposomes	Lactoferrin	Hyaluronic acid-coated liposomes	In vitro and in vivo	-Physical stability -Prolonged release of the drug -Biocompatible without any sign of ocular irritation or cytotoxicity	López-Machado A et al. [83]
Niosomes	Tacrolimus	Hyaluronic acid-coated niosomes	In vivo (rabbit model)	-Prolonged residence time of the drug -Enhancement in transcorneal permeability	Zeng W. et al. [86]
Nanoparticles	Tacrolimus	Gellan gum nanoparticles	In vitro and in vivo (rabbit model)	-Prolonged drug release throughout 12 h and higher precorneal retention compared to tacrolimus solution -Amelioration in DED symptoms in rabbits	Modi D et al. [90]
	Cerium oxide	Water-soluble glycol chitosan nanoparticle	In vitro and in vivo (murine model)	-No cytotoxic effects -Improvement in dry eye disease models by stabilizing the tear film, promoting and maintaining corneal and conjunctival cell growth and integrity	Yu F. et al. [96]
	Cyclosporine A	Lipid nanocapsule	In vitro and in vivo (rabbit model)	-Improvement in bioavailability and permeability -Amelioration in BUT, fluorescein staining, tear production, and histopathology tests	Zhang A. et al. [97]
Nanostructured lipid carriers	Cyclosporine A	Thiolated nanostructured lipid carrier	In vitro and in vivo (rabbit model)	-Higher concentration of CsA in aqueous, humor, tear, and eye tissues	Shen J. et al. [94]
Nanowafers	Dexamethasone	Polydimethylsiloxane nanowafers	In vivo (mice model)	-Preservation of corneal clarity -Decreasing expression of metalloproteinases and inflammatory cytokines	Bian F. et al. [98]
Dendrimers	Dexamethasone	Subconjunctival injectable gel based on G4-PAMAM dendrimer and hyaluronic acid	In vivo (rat model)	-Reduction in corneal inflammation more effective than with free-dexamethasone -Enhanced corneal clarity without causing an increase in intraocular pressure levels	Soiberman et al. [102]
Hydrogels	Cyclosporine A	Nanostructured poly (2-hydroxyethyl methacrylate) (p-HEMA) hydrogels containing microemulsions or micelles of Brij 97	In vitro	-Sustained and controlled release (20 days) of drugs. -Resistance after exposure to all the relevant processing conditions	Kapoor Y. et al. [123]
	Hyaluronic acid	Soft hydrogels	In vivo (canine model)	-Biocompatibility -In combination with CsA, improved clinical signs in more than 65% of dog patients previously unresponsive to Cyclosporine treatment	Yu Y. et al. [104]
Drug-eluting contact lenses	Hyaluronic acid	Contact lenses prepared by soaking method or direct entrapment method	In vivo (rabbit model)	-Safe profile -Increased residence time of hyaluronic acid with lenses compared to conventional eye drop treatments	Maulvi FA et al. [106]
Nanogels	Lysine hydrochloride	Carbonized nanogels	In vitro and in vivo (rabbit model)	-High biocompatibility -Reduction in the therapeutic dose and extended dosing interval	Lin PH et al. [111]
Nanozymes	Cerium oxide	Cerium oxide nanozyme combined with branched poly(ethylene imine)-graft-poly(ethylene glycol)	In vitro and in vivo (murine model)	-Biocompatibility -Antioxidant activity -In vivo reduction in corneal epithelial defects and increased goblet cells	Zou et al. [113]
	Dual-atom (Fe-Mn)	Fe and Mn atoms embedded in N-doped carbon material and modified with hydrophilic polymer	In vitro and in vivo (murine model)	-Inhibition of NLPR3 inflammasome activation -Antioxidant activity -Reduced corneal opacity -Reduced fluorescein staining	Chu et al. [112]

**Table 3:** Nano-based drug delivery systems approved for dry eye disease.

Trade Name	Therapeutic Agent	Nanosystem	Outcomes	References
Restasis^®^	Cyclosporine A	0.05% oil-in-water anionic nanoemulsion. Polysorbate 80 as surfactant and castor oil as solubilizer	-Increase in conjunctival goblet cell density -Reduction in punctate fluorescein staining -Amelioration in symptoms of blurred vision -Safe profile of action	Sall K. et al. [124] Stevenson D. et al. [142]
Lacrinmune^®^	Cyclosporine A	Oil-in-water emulsion	-The composition is similar to Restasis^®^ but with the addition of sodium hyaluronate, which allows an increased viscosity and a prolonged retention time on the ocular surface	Lv Z. et al. [6]
Cationorm^®^	Lipids, glycerol	Nanoemulsion	-Effective in evaporative and non-evaporative DED -Excellent safety profile -Transient blurred vision observed in some patients	Fogagnolo P. et al. [143]
Ikervis^®^	Cyclosporine A	Cationic emulsion 0.1%	-Improvement in global symptom and corneal staining scores at 6 months -Greater bioavailability of CsA to the ocular surface compared to anionic emulsion	Baudouin C. et al. [144] Lallemand F. et al. [145]
Cyclokat^®^	Cyclosporine A	Cationic emulsion 0.1%	-Improvement in signs and symptoms in patients suffering from moderate-to-severe dry eye syndrome	Buggage RR et al. [129]
Cequa^®^ (OTX-101 0.09%)	Cyclosporine A	Aqueous nanomicellar solution	-Improved corneal and conjunctival staining -Good tolerability -Rapid onset of action	Goldberg DF et al. [146] Mandal A. et al. [77]
Vyseo^®^	Vitamin A and vitamin E	Phospholipid liposomal spray	-Useful for the treatment of patients with mild-to-moderate evaporative DED	Nagai N. et al. [74]
Clarimist^®^	Vitamin A palmitate and vitamin E	Liposomal spray		
Tears Again^®^	Hyaluronic acid	Phospholipid liposomal spray	-Improvement in tear film stability, symptoms, and visual acuity	Craig JP et al. [135]
Lacrisek^®^	Vitamin A palmitate and vitamin E	Liposomal spray	-Local vitamin A supplementation is useful in improving goblet cell density and epithelial health	Fogagnolo P. et al. [138]
Artelac Rebalance^®^	Vitamin B12	Liposomal spray	-2-months application in mild-to-moderate dry eye cases resulted in a reduction in ocular inflammation parameters, ocular surface damage, and subjective discomfort symptoms -High tolerability and satisfaction -No adverse events reported	Versura P. et al. [147]
Vidisc^®^gel	Polymerizate acrylic acid	Hydrogel	-Longer viability compared to other tear substitutes -Well tolerated and effective	Marquardt R. [148]
Hylo^®^gel	Hyaluronic acid 0.2%	Hydrogel	-Significant improvements in objective findings and subjective symptoms when used as a lubricant after penetrating keratoplasty	Pattmöller M. et al. [149]
GelTears^®^	Carbomer 980	Hydrogel	-Extended contact of solutes or suspended solids with the corneal surface	Wilson CG et al. [150]
Viscotears^®^	Carbomer 980	Polyacrylic acid 0.2% hydrogel	-Local tolerability upon instillation -Improvement in subjective symptoms and objective test results after 30 days of treatment	Bron AJ et al. [151]
Eysuvis^®^ (KPI-121 0.25%)	Loteprednol etabonate	Nanoparticles coated with Poloxamer 407	-Good tolerability -No significant increase in intraocular pressure after 2-week treatment	Korenfeld M. et al. [140] Venkateswaran N et al. [139]
Cyclasol^®^	Cyclosporine A	Nonaqueous solution without water, oil, surfactants, or preservatives	-Enhanced bioavailability and efficacy -Early therapeutic effects on the ocular surface -Safety, tolerability, and comfort profile	Akpek EK et al. [141] Wirta DL et al. [152]

## Data Availability

Data sharing not applicable. No new data were created or analyzed in this study.
